# Rationale and design of the Chinese Atrial Fibrillation Registry Study

**DOI:** 10.1186/s12872-016-0308-1

**Published:** 2016-06-07

**Authors:** Xin Du, Changsheng Ma, Jiahui Wu, Songnan Li, Man Ning, Ribo Tang, Xueyuan Guo, Deyong Long, Ronghui Yu, Caihua Sang, Chenxi Jiang, Ting Zhang, Jianhong Pan, Xiaohui Liu, Jianzeng Dong, Gregory Y. H. Lip, on behalf of the CAFR Investigators, Hongwei Li, Ming Yang, Hong Jiang, Xiaozhong Zhang, Jie Jiang, Ping Zhang, Qijun Shan, Quan Fang, Shuwang Liu, Xian Wang, Yang Wu, Qi Hua, Xingshan Zhao, Lihua Shang, Haibin Zhang, Aijuan Liu, Jinping Wang, Ruijie Li, Zhengyan Zhu, Xuemei Peng, Lin Pi, Lantang Han, Weiju Li, Ling Sun, Chunhong Li, Xudong Chen, Buxing Chen

**Affiliations:** Beijing Anzhen Hospital, Capital Medical University, No 2 Anzhen Road, Chaoyang District, Beijing, 100029 China; Peking University Clinical Research Institute, Beijing, China; University of Birmingham Institute of Cardiovascular Sciences, City Hospital, Birmingham, UK

**Keywords:** Atrial fibrillation, Registry study, China

## Abstract

**Background:**

Robust data on the contemporary management of atrial fibrillation (AF) patients in China are limited. Importantly current practice in AF management has changing dramatically in recent years. Data from a large registry study will enable us to evaluate the uptake and outcomes with different therapies in a large Chinese AF population.

**Methods/Design:**

The Chinese Atrial Fibrillation Registry study (CAFR) aims to enroll 20,000 consecutive atrial fibrillation (AF) patients from 32 tertiary and non-tertiary hospitals in Beijing, China, and follow up these patients every 6 months until 2020. Key data collected includes basic sociodemographic information, symptoms and signs, medical history, results of physical examination and laboratory test, details of treatments and personal insurance status. For patients who consent, 5 ml of blood sample will be stored at −80 °C for future analyses of biomarkers. At each 6 month follow up visit, data relating to clinical outcomes will be collected. Data from a randomly selected 10 % of patients will be internally validated with their raw source data. Ischemic stroke events will be adjudicated by an independent endpoint committee.

**Discussion:**

CAFR will be one of the largest registries of Asian AF patients (and the largest in Chinese AF patients), as well as providing the longest follow up. This study would provide a valuable opportunity for ‘real world’ clinical epidemiology with insights into the uptake (and outcomes) of contemporary AF management.

**Trial registration:**

Chinese Clinical Trial Registry ChiCTR-OCH-13003729. Registered 22 October 2013.

**Electronic supplementary material:**

The online version of this article (doi:10.1186/s12872-016-0308-1) contains supplementary material, which is available to authorized users.

## Background

Atrial fibrillation (AF) is the most common sustained cardiac arrhythmia and carries an increased risk of stroke, hospitalization, and mortality [1, 2]. It is estimated that at least 10 million patients in China suffer from AF [3]. With the ageing population and associated prevalence of other cardiovascular diseases, the burden of AF is projected to increase even further in China.

Although AF has been identified as a major risk factor for stroke, recommendations on anticoagulation therapy in Asian population are inconsistent in several aspects, including criteria for anticoagulation and the intensity of warfarin use. There is a perception that Asians are more prone to bleeding (especially intracranial bleeding) on warfarin [4, 5] and some guidelines even recommend that the Asian population should target a lower international normalized ratio (INR) range (eg. 1.6–2.6) when warfarin is used [5, 6]. Despite limited evidence supporting this practice, Chinese doctors tend to be conservative when prescribing oral anticoagulation (OAC) therapy for AF patients, reflected by an extremely low usage of OAC. When warfarin is prescribed, a lower INR is usually targeted. Indeed, limited data on quality of anticoagulation control (as reflected by time in therapeutic range, TTR) are available from Asian cohorts, despite TTR being closely related to the efficacy and safety of warfarin [7].

Thus, real world data may provide additional evidence to guide our practice when evidence from randomized trial is less likely to be obtained. In addition, the treatment of AF is changing in many ways in China. For example, catheter ablation is provided in majority of tertiary hospitals despite the shortage of evidence supporting its effectiveness on patient centered outcomes. Non-Vitamin K antagonist oral anticoagulants (NOACs) are increasingly prescribed whilst data of effectiveness and safety of these agents is more limited among Chinese population. Left atrial appendage occlusion was introduced in China in the last 2 years and real world data would be valuable in evaluating the effectiveness of this new technique.

Third, we recognize that genetic susceptibility contributes to the development of some diseases and the patients’ response to therapy [8]. Indeed, some patients with paroxysmal AF will not progress to persistent AF for many years while others progress more quickly. Stroke also happens in some AF patients without any known risk factors. Incorporating phenotype and genotype studies will provide the opportunity for better understanding of AF as a disease entity and for personalized treatment.

The Chinese Atrial Fibrillation Registry study (CAFR) is a prospective registry study launched in August 2011 and is still ongoing. Herein we reported the rationale, design of this study and current status of the database.

### Research objectives

CAFR aims to provide evidence for clinical practice based on real world data. The main objectives are as follows: (i) to compare and validate current risk scores for stroke and bleeding risk assessment in Chinese AF patients; consequent upon this, to improve current risk prediction models using phenotype and genotype data; (ii) to study factors associated with anticoagulation under-use, variation in stroke prevention practice and progress over the study period in typical tertiary and non-tertiary hospitals; and (iii) to report on safety and long term effectiveness of AF ablation therapy, and compare the effectiveness of ablation and medical therapy in reducing death and thromboembolic events, using propensity score matched cohorts.

The following additional exploratory analyses will also be considered: (i) description of effectiveness and safety of NOACs and left atrial appendage closure among the Chinese AF population; and (ii) assessment of biomarkers associated with AF progression from paroxysmal to persistent AF, and development of complications.

## Methods/Design

### Study setting

Thirty two tertiary and non-tertiary hospitals at where the majority of AF patients in Beijing are managed, have agreed to participate in this study. The characteristics of participating hospitals are listed in Table 1.

**Table 1:** Characteristics of participating hospitals

	Tertiary hospitals	Non-tertiary hospitals
No of hospitals	20	12
No. of beds for cardiovascular disease, Median (IQR)	78 (59–120)	46.5 (12–80)
AF ablation facilities	18/20 (90 %)	0/12 (0 %)

### Patient inclusion and exclusion criteria

Patients aged 18 years or older with a documented AF as confirmed by 12 leads ECG, pacemaker/ICD electrocardiogram, or Holter ECG (duration of AF episode least 30 s) are eligible for this study. Patients with transient AF caused by reversible cause (e.g., cardiac surgery, pulmonary embolism, untreated hyperthyroidism) or combined with other serious diseases with a life expectancy < 1 year were excluded. All patients with the diagnosis of AF who are under the care of cardiologists or general physicians in the participating hospitals, and provided informed consent will be enrolled, from both outpatient and inpatient settings. Each hospital maintained an admission record to provide evidence of whether there was selection bias, by comparing the mean age, sex and comorbidities of enrolled and non-enrolled patients. Enrollment will cease when the target of 20000 patients is achieved.

### Clinical data collection

Key data elements and definition of each variable were in line with the ACC/AHA recommendation on AF clinical data standards [9] and international peer studies [10] to facilitate cross-comparison of results between different registries. The following data were collected for each patient enrolled: basic socio-demographic information, symptoms and signs relating to AF, medical history, results of physical examination and laboratory test and patient self-reported quality of life (using a validated Chinese version of AFEQT questionnaire). For patients undergoing ablation therapy, detailed procedure related information is also collected, including the pathway of ablation, achievement of conduction block, time consumed and peri-procedure complications.

Each enrolled patient will be followed up every 6 months by trained staff, at outpatient clinic or through telephone interview. Information relating to medical therapies, health-care utilization, and clinical outcomes will be collected during follow up. Definition of the major outcome events are provided in Table 2. Every patient will be followed up until 2020. The questionnaires used for data collection in study are provided in appendix. All the data were entered into a specific electronic data capture system.

**Table 2:** Outcomes collected and associated definitions

Outcomes	Definitions
Primary outcome	
Composite outcome of any of the following events: all-cause mortality, non-fatal ischemic stroke and peripheral embolism.	All-cause mortality: All deaths regardless of etiology
	Non-fatal Ischemic stroke: Documented stroke or cerebrovascular accident consisting of acute loss of neurological function caused by an ischemic event with residual symptoms at least 24 hours after onset.
	Peripheral embolism: Abrupt vascular insufficiency associated with clinical and radiological evidence of arterial occlusion in a vascular bed other than the cerebrovascular system in the absence of other likely mechanisms (e.g., atherosclerosis).
Secondary outcomes	
Component of the primary composite outcome including: 1) all-cause mortality; 2) non-fatal stroke or peripheral embolism.	As above
Intracranial hemorrhages	Bleeding into or around the brain, including 1) Hemorrhagic conversion of a primary ischemic stroke; 2) Subarachnoid hemorrhage; 3) Intra-cerebral hemorrhage; 4) Other (including subdural and epidural hematomas);
Major bleeding	Bleeding leads to 1) Transfusion of at least 2 units of whole blood or erythrocytes; 2) Requiring hospitalization or surgery; 3) Resulting in permanent disability; 4) Involving a critical anatomic site (retroperitoneal, pericardial, intra-spinal, intracranial, non-traumatic intra-articular, or intra-ocular bleeding associated with abrupt deterioration of visual acuity).

### Blood sample for future analysis

Five milliliter venous blood sample will be collected from patients with consent. The sample will be centrifuged. Plasma and blood cells will be separately stored at −80 °C refrigerators. Standard operation procedures for blood collection, processing and archiving are in line with the International Society for Biological and Environmental Repositories’ Best Practices for Repositories [11]. FreezerPro® is used for web-based management of frozen samples and information of sample will be linked to corresponding clinical data.

### Data management and quality control

All clinical data will be captured via a web-based electronic data capture (EDC) system. Investigators will enter and edit the data via a secure network, with secure access features. Variables related to the answer of main aims are mandatory to be entered. The database also had function of range and logic check. Source material will be kept in hospital for further monitoring and auditing. The quality control team will review all the data collected case by case and send queries when applicable.

A random selection of 10 % cases will have their raw data validated, for baseline data collection and during each follow up. All reported ischemic stroke and peripheral embolism events will be adjudicated by an independent endpoint committee.

### Ethical considerations

Ethics approval was obtained from the Human Research Ethics Committees at Beijing Anzhen Hospital. Ethic review boards in each participating hospital agreed their participation. The data collected will be held centrally in a secure database, only de-identified information can be used for analysis. Informed consent from individual patients was sought for their agreement to participate in long-term follow-up and usage of their blood sample for further analysis. The study was performed according to the ethical principles for medical research involving human subjects specified in the Declaration of Helsinki.

### Statistical analysis

The CHADS_2_ schema and CHA_2_DS_2_-VASc schema will be compared and validated in these Chinese AF patients, after excluding patients receiving anticoagulation therapy or ablation therapy during follow up. Logistic regression will be used to evaluate the predictive value of the various risk schemata in discriminating between patients who develop the outcome of interest and those who do not. The c-statistic will be estimated to determine the predictive ability for stroke of each schema. The 95 % confidence interval will be estimated for the c-statistic using a non-parametric bias-corrected bootstrapping method. To assess the effect of individual risk factors on the occurrence of events, multivariable logistic regression will be used with the baseline covariates as independent variables. According to the result of the multivariable logistic regression analysis, the score for each risk factor will be adjusted for the best fitted classification schema. If necessary, new risk factors may also be added to generate a new stroke risk classification schema for Chinese AF population. The new schema will be validated on the validation population and the C-statistics will be calculated using the new risk schema. In addition, the net reclassification index will also be used to evaluate predictive value of the prognostic score.

To compare the ablation and non-ablation therapies, propensity score matching for receiving catheter ablation will be calculated for each patient based on a multivariable logistic regression model. Baseline characteristics variables which are assumed to be associated with the probability of having catheter ablation will be included in the model as independent variables. Based on the propensity scores, ablation and non-ablation patients will be matched on a 1:1 basis with a combination of the nearest neighbor algorithm and caliper algorithm [12]. For each ablation patient, a non-ablation patient with the smallest propensity score difference, and smaller than 0.02, will be matched without replacement. Patients not matched will be excluded from the analysis.

Unadjusted event rates for the composite primary outcome of time to first all-cause mortality, stroke and peripheral embolism for the propensity-matched ablation and non-ablation groups will be estimated using the Kaplan-Meier method and compared between groups with the log-rank test. As sensitivity analyses, the inverse probability of treatment weighting using propensity score will also be used to compare the survival distributions between ablation and non-ablation group.

### Sample size calculation

For the objective of developing the optimal risk prediction model for Chinese AF population, a minimum of 200 events (i.e. 10 times of the variable number) are required to accommodate 20 variables in the prediction model. The analysis is planned to be done in 2015 to accumulate enough events for analysis. For that purpose, about 5000 patients without anticoagulation therapy are required for an evenly splitted training cohort and validation cohort. For the objective of evaluating the effectiveness of ablation therapy, the following assumption is made: the accumulate ischemic stroke and intracranial hemorrhage is 10 % in optimally anticoagulated patients and 7 % in ablation patients in five years, 1800 matched patients in each arm are required to provide 90 % power to detect the difference (α = 0.05). As those two groups of patients are quite different, we amplified the sample size by 3 times to guarantee enough matched patients. In total, 16000 patients are required for the main analyses and considering a possible 20 % withdraw and loss of follow up, we up round the sample size to 20000.

## Results

### Current status and data quality

17467 patients had been enrolled as at June 30, 2015. The majority (84.0 %) came from tertiary hospitals where most AF patients are managed. The missing rates for the key variables are listed in Table 3. There are no missing data among the mandatory variables which is controlled by the EDC system. Out of range variables were very rare (<0.1 %) for those with range check. Patient follow up rates at 6, 12, 18 and 24 months are 93, 91, 87 and 85 % respectively.

**Table 3:** Variables collected in the CAFR study and missing rate for each key variables

Variables	Number of variables currently collected	Missing rate (%)
Age or Date of Birth	17467	0 %
Sex	17467	0 %
Date of initial register	17467	0 %
Type of AF	17340	0.73 %
Systolic blood pressure	17291	1.01 %
Height	15845	9.29 %
Body weight	15964	8.60 %
History of alcohol-drinking	17250	1.24 %
History of hypertension	17331	0.78 %
History of diabetes mellitus	17331	0.78 %
History of hyperlipidemia	17288	1.02 %
History of heart failure	17331	0.78 %
History of myocardial infarction	17328	0.80 %
History of coronary artery disease	17330	0.78 %
Signs of heart failure	17329	0.79 %
Hemoglobin level	13552	22.4 %
Creatinine level	14264	18.3 %
Fasting blood glucose	13718	21.4 %
Total cholesterol	13587	22.2 %
Is thyroid function within normal range	12367	29.2 %
Left ventricular ejection fraction	13568	22.3 %
History of direct current cardioversion	17268	1.14 %
History of radiofrequency ablation	17332	0.77 %
History of ischemic stroke/transient ischemia attach/ peripheral arterial thromboembolism	17338	0.74 %
History of intracranial hemorrhage/other major bleeding	17335	0.76 %
Use antiarrhythmic agents	17334	0.76 %
Use ventricular rate control agents	17332	0.77 %
use antithrombotic agents	17333	0.77 %
Warfarin	17281	1.06 %
Use Angiotensin converting enzyme inhibitors/angiotensin receptor blockers	17281	1.06 %
Use Statins	17283	1.05 %

## Discussion

This large, contemporary, longititudinal study of Chinese AF patients will provide a unique opportunity to answer many clinical questions and to expand our knowledge of AF development and treatment.

**Table 4:** Atrial fibrillation registry studies worldwide

Studies	Participating centers	Timeline	Follow-up	Sample size	Main purpose
RHYTHM-AF [18]	10 countries, 175 centers, mostly European countries	May 2010 ~June 2011	60 days	3940	To describe treatment patterns and short term outcomes related to cardioversion.
GARFIELD [19]	50 countries in Europe, Asia-Pacific, Central/South America, and Canada	Started in December 2009, with a planned recruitment period of 4 years	2-year	55,000	To evaluate the management and outcomes of patients with newly diagnosed non-valvular AF at risk for stroke.
GLORIA-AF [20]	Nearly 50 countries in Asia, Europe, North America, Latin America and Africa/Middle East	Phase I: May 2011- January 2013.	Phase II: 2 years	56,000	To investigate patient characteristics influencing choice of antithrombotic treatment and to collect data on outcomes of antithrombotic therapy
		Phase II: November 2011	Phase III: 3 years		
		Phase III: early 2014			
J-RHYTHM [21]	158 institutions in Japan	January to July 2009	2-year	7937	To determine the appropriate INR for Japanese AF patients
PINNACLE-AF [22]	In the United states	October 2011	-	>300000	Quality improvement
The Atrial Fibrillation Ablation Pilot Study [23]	72 centers in 10 European countries	October 2010	1 year	1410	to describe the clinical epidemiology and treatment of patients undergoing AF ablation
ORBIT-AF [24]	200 US outpatient practices	June 2010 to August 2011.	≥2 years	10097	To characterize treatment and outcomes of patients with AF

*Abbreviations*: *RHYTHM-AF* International Registry on Cardioversion of Atrial Fibrillation, *GARFIELD* Global Anticoagulant Registry in the FIELD, *GLORIA-AF* Global Registry on Long-Term Oral Antithrombotic Treatment in Patients with Atrial Fibrillation, *J-RHYTHM* Japanese Rhythm Registry, *PINNACLE-AF* The nationwide US Practice INNovation And Clinical Excellence Registry, *ORBIT-AF* The Outcomes Registry for Better Informed Treatment of Atrial Fibrillation

The CAFR study is important in several respects. First, systematic observational data can be generated from this registry study, which is especially valuable given that evidence for Chinese AF patients is limited. Results from randomised clinical trials remain the most robust evidence for clinical decision making, these can be expensive and time consuming to be feasible in many scenerios. Second, treatment of AF is changing dramatically. For example, ablation therapy is increasingly widely provided while there is short of evidence to support the practice, especially in the Chinese AF population. NOACs have proved their efficacy in stroke prevention [13–17] but whether the efficacy observed in randomized trials translates into effectiveness and safety in Chinese AF patients need to be evaluated in real world studies. Third, the CAFR study provides a good opportunity to compare treatment and response variation among AF populations in China, for comparison with different countries. Currently, several other large AF registries are ongoing. The main characteristics of large size registry studies are listed in Table 4.

The CAFR study is unique compared with other AF registry studies in the following respects: (i) CAFR is the largest real world study among Asian AF patients and will generate invaluable data for this patient group; (ii) CAFR is a longitudinal study which enable evaluation of long term effects of specific therapies; and (iii) A high proportion of patients had their blood sample taken, which enables investigation of genetic and environment interactions in the developement and treatment of AF.

### Limitations

Participating hospitals of this study are all hospitals in urban and semi-urban area of Beijing, the capital of China. However, about half of the registied patients come from all around the country, which increases the representativeness of the study population. In addition, the majority of patients are registred from tertiary hospitals, but this reflects current practice of Chinese medical care as the mechanisms of referral are not fully established and patients usually go direct to terriary hospitals without referral from primary health care providers. Despite these limitations, we feel that we are unlikely to overtly bias conclusions for the prespecified main analysis of this study.

## Conclusion

CAFR will be one of the largest registries of Asian AF patients (and the largest in Chinese AF patients), as well as providing the longest follow up. This study would provide a valuable opportunity for ‘real world’ clinical epidemiology with insights into the uptake (and outcomes) of contemporary AF management.

## Abbreviations

AF, atrial fibrillation; CAFR, Chinese atrial fibrillation registry; EDC, electronic data capture; INR, international normalized ratio; NOAC, non-vitamin K antagonist oral anticoagulants; OAC, oral anticoagulation; TTR, time in therapeutic range

## Additional file

Additional file 1: Ethics committees of CAFR. (DOCX 20 kb)
