# Supplementary material for: Rationale and design of the Chinese Atrial Fibrillation Registry Study
Source: BMC Cardiovasc Disord. 2016 Jun 7;16:130. doi: 10.1186/s12872-016-0308-1 (PMC4897860; doi:10.1186/s12872-016-0308-1)
Supplement: Additional file 1: — Ethics committees of CAFR. (DOCX 20 kb) [file 12872_2016_308_MOESM1_ESM.docx]

**Ethics committees of CAFR**

Figure Legend: Ethics approval of CAFR was obtained from the Human Research Ethics Committees at Beijing Anzhen Hospital. Ethic review boards in each participating hospital agreed their participation. The individual ethics committees of all hospitals included in CAFR are named in the supplelmentary file.

**Tertiary Hospitals**

1. Beijing An-Zhen Hospital

Wei Yongxiang, Jin Chunming, Chen Fang, Zhou Yujie, Zhang Hongjia, Gao Hai, Liu Yuyong, Yu Yang, Lv Biao, Liu Shuang, Liu Jing, Deng Liqiang

2. Peking Union Medical College Hospital

Director: Shen Keng

Vice Director: Zhai Xiaomei

Secretary: Huang Hui

Committee: Bai Ye, Chen Xiaowei, Fang Ligang, Feng Feng, Gan Jia, Hong Xia, Li Xuemei, Li Jingnan, Li Longcheng, Lin Yansong, Liu Dawei, Liu Xinyan, Mei Dan, Ning Xiaohong, Pan Lingya, Wang Huanling, Wu Zhihong, Yin Jia, Yu Kang, Zhang Xuan, Zhu Chaohui, Zhu Yicheng, Cao Wenli, Wang Yanwen

3. Chinese PLA General Hospital

Director: Shi Qinglong

Vice Director: Yan Junlan, Wang Rui, Zhang Yong, Chen Qian

Committee: Wang Zongze, Wang Peilan, Wang Jin, Lu Yanping, Tian Hui, Chu Xiangyang, Liu Guangdong, Liu Ping, Zhang Li, Zhang Yu, Zhang Bao Zhu, Li Zhiwen, Fan Zhen, Zheng Qiufu, He Tao, Ke Bin, Cao Xiutang, Huang Feng

4. Chinese Civil Aviation General Hospital

Director: Li Songlin

Vice Director: Xu Xianfa, Wan Gang

Secretary: Gao Li

Committee: Wang Shuming, Peng Dingqiong, Ji Hanhua, Zhao Xu, Gao Li, Jiang Jufang, Xu Xiangqun, Wang Lijun, Wang Chenlong, Huang Peng, Li Jingmin, Li Yumin, Li Jiaxin, Yang Yue, Wang Hong, Hu Qingjun, Wang Qi, Zhao Xuezeng, Lu Binghuai, Li Qian, Li Qingyan, Zhang Tianli, Cong Yali, Zhao Liyan, Zhao Anping, Shi Liying

5. Beijing Guang’anmen Hospital

Director: Zhang Yunyun

Vice Director: Tong Xiaolin

Secretary: Lian Fengmei

Committee: Yin Haibo, Gu Lizhen, Shen Ruiying, Zhao Jun, Yang Herong, Wang Yinghui, Piao Bingkui, Zhang Jumin, Hu Jingqing

6. Beijing Tong-Ren Hospital

Director: Wang Ningli

Vice Director: Zhang Luo, Huang Zhigang

Committee: Yang Jinkui, Jin Xiaobing, Wang Yan, Yu Zhengya, Zhang Qingyu, Wang Jiawei, Zhao Xiuli, Jiao Yonghong, Cai Ying

7. Beijing Jishuitan Hospital

Zhao Xiaolan, He Liang, Jiang Xieyuan, Ma Yuhua, Guo Yuan, Lan Yu, Wang Manyi, Wu Chengai, Xia Guoguang, Zhang Guoying, Zhen Jiancun, Guo Jianguang, Huang Jian, Liu Wei, Zhao Danhui, Sun Lei, Zhang Huan, Zhang Wei

8. Beijing Friendship Hospital

Director: Zhang Jian

Vice Director: You Hong

Secretary: Cui Yan

Committee: Shi Limin, Wang Huiying, Ma Li, Sun Yan, Deng Liqiang, Jiang Huan

9. The 307^th^ Hospital of Military Medicine Sciences

Zhang Xiaozhong, Xue Jian

10. Beijing Dongzhimen Hospital

Wang Xian, Zhu Haiyan, Zheng Liang, Sun Mengqiong, Xuan Changbo

11. Peking University People’s Hospital

Li Xuebin, Zhang Ping, Duan Jiangbo

12. Peking University Third Hospital

Gao Wei, Li Haiyan

13. Beijing Xuan-Wu Hospital

Hua Qi, Sun Xipeng

14. Beijing Chao-Yang Hospital

Chen Yong, Yang Xinchun, Wu Anshi

15. China-Japan Friendship Hospital

Jiang Hong, Zhang Shiyu, Wang Nan

16. Beijing Tian-Tan Hospital

Chen Buxing, Lin Tao, Xu Xiaowei

17. Beijing Hua-Xin Hospital

Shang Lihua, Zhang Tao, Tan Pengjin

18. Beijing Fu-Xing Hospital

Han Ling, Zhao Yan

19. Beijing University of Chinese Medicine East Hospital

Wu Yang, Kuang Wu, Zhang Jingqian

20. Peking University First Hospital

Jiang Jie, Huo Dongbo

**Non-tertiary Hospitals**

21. Beijing Lu-He Hospital

Director: Quan Junya

Vice Director: Zhao Liang, Gao Lejie

Committee: Gao Jiayi, Chen Shicai

Counselor: Li Yiting, Yu Zhenshan, He Yongfu

22. The Hospital of Shun-Yi District, Beijing

Director: Wang Fei

Vice Director: Zhao Yuehua

Committee: Zhu Zhengyan, Chen Xisheng, Feng Kai, Liu Xiaomin, Kang Quanli, Luo Gongtang, Meng Jun, Zhou Degui, Jiang Ai’min, Wang Chaoshan, Li Huizhen, Liu Guangying, Li Xiaoqiang, Zhang Dangsheng, Wang Fei, Zhao Yuehua, Jiang Guifang, Guo Fuyong, Cai Luping

23. Beijing Ping-Gu Hospital

Director: Wei Guanglin

Vice Director: Wang Xiaoyong, Sun Qingyue

Committee: Wang Yuqing, Xiong Xiuhua, Zhao Mingze

24. The First Hospital of Fang-Shan District, Beijing

Director: Li Jingrong, Xu Xueyu

Vice Director: Qin Fujun

Secretary: Chen Jinmei

Committee: Zhang Wuning, Guo Ye, Zhang Leilei, Zhang Xuegong, Zhang Shixiang, Wang Lijing, Zhang Zhenzong, Yang Linchun, Wang Jian, Wang Zijun, Huang Zhandong, Li Jianhua, Zhou Qiufeng, Han Wenyan, Wang Yonggui

25. People’s Hospital of Beijing Da-Xing District

Huang Dongming

26. Beijing Mi-Yun Hospital

Liu Yajuan, He Dan

27. Peking University Hospital

Li Weiju, Cui Hengping

28. Beijing Chuiyangliu Hospital

Cai Hui, Sun Xinping, Gu Xian’en, Su Guang

29. Beijing Chang-Ping Hospital of Integrated Chinese and Western Medicine

Yan Qiuli, Zhang Dian

30. Health service centre of Wangsiying Community

Chen Xudong, Si Yang

31. Health service centre of Ganjiakou Community

Li Chunhong

32. Beijing Da-Xing Jiugong Hospital

Sun Ling, Bao Guoxia
